# Fat embolism in sickle-cell disease: A case report with literature review

**DOI:** 10.22088/cjim.14.1.143

**Published:** 2023

**Authors:** Ahmad M. Almatar, Kawther Kawther

**Affiliations:** 1 Department of Neurology, King Fahad Hospital of the University, Khobar, Saudi Arabia; 2 Neurocritical Care, King Fahad Hospital of the University, Khobar, Saudi Arabia

**Keywords:** Cerebral fat embolism, Sickle-cell disease, Starfield appearance, Non-traumatic cerebral fat embolism

## Abstract

**Background:**

Sickle cell disease (SCD) is associated with an under-recognized and well-known complication of bone marrow necrosis known as cerebral fat embolism (CFE).

**Case Presentation:**

We report a case of successfully treated SCD patient suffering from non-traumatic cerebral fat embolism (NCFE) who came with initial manifestation of localized pain followed by loss of consciousness and absence of cardiac shunt.

This was an unusual case of fat embolism in SCD patient with the absence of right-to-left cardiac shunt indicating the biochemical etiology. Patient initially showed localized pain followed by loss of consciousness, suggesting that any such initial manifestation in patient of SCD should be suspected for CFE. MRI served as an accurate diagnostic tool showing the characteristic neuroradiologic sign. Treatment with exchange-transfusion recovered the patient successfully.

**Conclusion:**

Overall, this case suggested that prompt diagnosis using MRI and treatment with RBC exchange could lead to better outcomes in SCD patients suffering from CFE.

Cerebral fat embolism (CFE) constitutes clinical signs and symptoms, which arise due to the release of fat emboli into the systemic circulation usually after traumatic long bone fractures. Patients classically present with a triad of respiratory failure, neurological deficits and petechial skin rash; however, these symptoms are not seen together in all the cases (1, 2). CFE has also been observed secondary to non-traumatic etiology such as steroid use, pancreatitis, sickle cell disease (SCD), etc (2). Here in the present study, our focus in on CFE associated with SCD. SCD is a hereditary hemoglobinopathy caused by a single nucleotide mutation in the gene coding for the β-globin chain. It is a systemic disease that is presented by recurrent vaso-occlusive crises, which is secondary to a change in the morphology of the red blood cells (i.e., sickling). This change in physical property of cells is triggered by various factors such as dehydration, exposure to cold or fever (3). The pathology of NCFE in SCD is not clear. It is thought to be produced by a vaso-occlusive crisis which affects the bone marrow causing its necrosis. The fat emboli are thought to arise due to this necrosis of bone marrow (1, 2, 4). To the best of our knowledge, there are no specific diagnostic criteria for this condition. Most of the literature covers diagnosis of traumatic CFE which has also been utilized to diagnose it in non-traumatic cases (2). Before the advent of MRI, the diagnosis was established solely based on clinical criteria and was confirmed only on autopsy (5-9). There are some case reports of the diagnosis being made based on the presence of fat in urine, bronchial lavage fluid, peripheral smear, fundoscopy of the eye or level of serum secretory phospholipase A2 (sPLA2) (4). 

However, after the successful implication of MRI for diagnosis, majority of the cases has (have) been confirmed by the starfield appearance in the brain MRI. Herein, this study reports a successfully treated case of nontraumatic cerebral fat embolism (NCFE) and used available studies to decipher various facts associated with the case.

## Case Presentation

Herein, we present a case of 37-years-old Saudi female suffering from SCD. She visited King Fahad University Hospital with the complaint of lower back pain and bilateral shoulder pain which initiated one day prior to her visit to hospital. Patient had a medical history of recurrent ER visits and admissions due to painful crises. Within two days of admission to the hospital, she started to show signs of confusion and her consciousness level dropped. However, there was no need of intubation. At this stage, patient was referred to the neurology and ICU teams for further care. It was found on assessment that the patient was not cooperative, Glasgow Coma Scale (GCS) was found to be 10/15 (E3, V3, M4) with asymmetrical spontaneous movement of lower extremities. CT / CTA reports were normal and lumbar puncture showed normal CSF parameters. Initial neurological examination showed grossly intact cranial nerves, hypotonic left lower limb, and normal tone on the opposite side. Power of the upper limbs was symmetrical at least 2/5, at least 1/5 on the left lower limb and at least 2/5 on the right lower limb. Reflexes were +2 all over except for left lower limb at +3. With respect to planters, there was equivocal response of the left and flexor response on the right side. Sensory, coordination and gait could not be assessed. 

**Figure 1:**
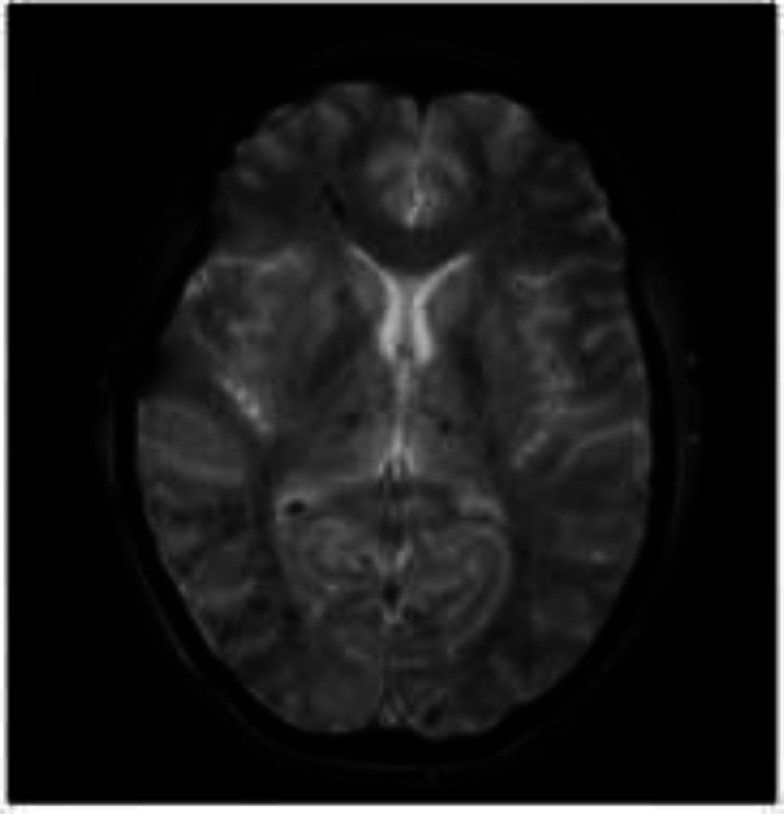
Brain MRI showing the starfield appearance

With this normal CSF and CT reports, it was thought that patient suffered from cerebral fat embolization, as all her symptoms were preceded by back and shoulder pain for which exchange transfusion was conducted. Her past medical history included bilateral hip replacement 1 year ago secondary to avascular necrosis for which she was admitted to surgical ICU as post-surgery care. Her treatment was as follows: Phlebotomy of 500 ml of blood followed by giving 500 ml 0.9 normal saline and 10 ml calcium gluconate 10% in 50 ml 0.9 normal saline. Then two packs of packed RBC's were transfused, each over 3 hours. Finally, 20 mg of furosemide were administered intravenously. After 4 days, the patient showed significant improvement in her consciousness level with no focal neurological deficit. However, there was psychomotor slowness in long term follow-up. MRI brain showed starfield appearance and incidental aneurysm in supraclinoid portion of right ICA (figure 1). Neurological examination after exchange transfusion showed GCS 15/15 and normal cognition but slow to respond. Other findings were as follows:- cranial nerves: intact; motor: normal tone, full power and symmetrical reflexes +2; planters: flexor response bilaterally; sensory: intact pin-prick. Coordination and gait were intact.

Values of other routine parameters like CBC, LFT and KFT have been provided as supplementary information. Echocardiogram showed no cardiac shunt. HPV B19 was not requested.

## Discussion

Very few reports are available in literature for the occurrence of CFE in patients with SCD^5^^- ^^13^ and more than that there are rare reports which indicate the successful treatment of CFE in SCD patients.^1^^,^^12^^,^^14^Moreover, CFE is potential under diagnosed or misdiagnosed. If CFE is associated with mild form of SCD, it could lead to late diagnosis. Various such reports have been found which show a higher death rate up to 40% in a relatively healthy patient when suffering from a painful crisis (15-17). One-third of these cases of sudden death on autopsy showed the presence of pulmonary fat emboli.^18^ CFE is also many times misdiagnosed for various other pathologies like sepsis, vasculitis, hypoxic-ischemic encephalopathy, acute hemorrhagic leukoencephalitis (19, 11). Herein, this study we have reported a case of CFE in a 36-year-old female with the diagnosis of SCD. The patient came with the localized pain which has been reported as the initial presenting symptom of this disease. Similar initial symptom of thigh pain was reported by Ositelu et al. in their study when the patient suffering from SCD initially came to the hospital and later was confirmed for CFE in brain MRI.^14^ Not only this study, our initial finding was in coherence with various other studies which have reported the first symptom in most of the patients as bone pain, whether back, abdomen, chest or lower limb 43.47% (10/23) (5, 6, 11-13, 20). Within two days, patient also started losing consciousness but did not require intubation, which is among the major cerebral symptom associated with CFE as evident by other studies (5-8, 11-13, 21, 22).

As discussed above, the pathology of CFE has yet not been completely deciphered; however, it is believed that it involves mechanical obstructions and biochemical injury (23, 24). It has been postulated in the mechanical theory that the formed fat microemboli enter into the venous sinusoids, thereby getting collected in pulmonary microvasculature. Sometimes, these migrate into the systemic circulation which occurs through pulmonary capillary bed or right-to-left cardiac shunts.^24^ On the other hand, it is postulated in biochemical theory that systemic release of free fatty acids occur through plasma mediators leading to inflammation and damage to endothelium (24). Since the patient in this case study was negative for shunting, it could be inferred that biochemical process might have mediated the development of cerebral fat emboli. As evident from the available literature and to the best of our knowledge, there are very few cerebral fat embolism cases in SCD reported which were without ACS or cardiac shunt which further raised concern that in such cases cerebral fat emboli might remain underdiagnosed (13). HPV B19 has also been established as a risk factor but it was not studied in this case (1). 

The diagnosis of NCFE requires proper clinical setting. There exists a scoring system for the diagnosis of CFE, but it has been found to show non-specific manifestations which create a high index of suspicion. However, it could be aided by clinical, laboratory and imaging findings for appropriate and accurate diagnosis (1). Laboratory findings usually indicate systemic inflammation and signs of organ damage (1). Peripheral smears sometimes show leucoerythroblastic picture. Diffused bilateral infiltrates are observed in chest radiography (23). In spite of all these diagnostics, MRI aids most accurately for the diagnosis of CFE wherein multifocal punctate infarcts are revealed along with microhemorrhages in a “starfield” pattern (13). In the present case, also brain MRI showed the presence of starfield appearance and incidental aneurysm in supraclinoid portion of right ICA, thereby ascertaining the diagnosis of CFE (figure 1). Among various cases reviewed by us, we found that in concurrence to our findings, the starfield appearance was observed in all the patients who had an MRI (17/17), of which most of them showed this pattern in the first scan itself (15/17) (5-8, 11-13, 21, 22). Usually, the treatment of FES includes supportive care and emergent red cell exchange transfusions. It has been found that early initiation of exchange transfusion could lead to break in sickling cycle and further halt the necrosis of bone marrow and fat embolism which improves the disease prognosis (12). In addition,it was observed in this patient that exchange transfusion improved the condition of patient gradually.

In conclusion patients with SCD have been found to suffer from a rare syndrome, i.e., NCFE which is preceded by events such as vaso-occlusion and localized or dispersed pain. However, there are very few reports for this condition due to poor diagnosis of the syndrome. The present case study showed that any painful crises could be an initial presentation for CFE in SCD patients. Moreover, patients of SCD who show changes in the mental status even in the absence of right-to-left cardiac shunt should also be suspected for cerebral fat emboli. Diagnosis using MRI conducted promptly in such patients followed by immediate treatment with red cell exchange transfusion could help in the successful treatment of such patients. This strategy would not only reduce the morbidity and mortality in these patients but also preserve functionality with better health outcomes.
